# Occurrence of *Giardia duodenalis* assemblages in farmed long-tailed chinchillas *Chinchilla lanigera* (Rodentia) from Romania

**DOI:** 10.1186/s13071-018-2652-8

**Published:** 2018-02-07

**Authors:** Călin Mircea Gherman, Zsuzsa Kalmár, Adriana Györke, Viorica Mircean

**Affiliations:** Parasitology and Parasitic Diseases Department Cluj-Napoca, University of Agricultural Sciences and Veterinary Medicine Cluj-Napoca, Faculty of Veterinary Medicine, Cluj-Napoca, Romania

**Keywords:** Farmed long-tailed chinchilla, *Chinchilla lanigera*, *Giardia duodenalis*, Prevalence, Assemblages, Romania

## Abstract

**Background:**

*Giardia duodenalis* is a parasitic protist that infects a large number of species, being localized in the small intestine. Two of the eight recognized assemblages have zoonotic potential, but studies regarding their distribution in less important pet or farm species are scarce. Of these species, the long-tailed chinchilla is a host for *Giardia* spp., although data on the spread of infection and assemblages involved are confined. The present work aimed to determine the prevalence of *Giardia* infection and assemblage identification in farmed chinchillas in Romania. A total of 341 fecal samples were collected from 5 farms and microscopically examined using flotation test based on saturated sodium chloride solution. DNA from all positive samples was extracted and identified by PCR targeting the *gdh* gene.

**Results:**

The overall prevalence of *Giardia* infection was 55.7% (190/341); there was no statistically significant difference (*P* = 0.25) in prevalence between young animals (58.8%) and adults (52.6%). Assemblages B (151/190), D (33/190) and E (6/190) were identified. Among assemblage B, sub-assemblages BIII (6/151) and BIV (145/151) were determined.

**Conclusions:**

This study demonstrates that *Giardia* spp. infection is highly prevalent in farmed chinchillas from Romania, and the sub-assemblages identified are potentially zoonotic.

## Background

The genus *Giardia* contains six species of aerotolerant anaerobic enteric protozoan parasites isolated from mammals, birds and amphibians [1–4]. Of all these species, three infect mammals, *Giardia muris* and *G*. *microti* in rodents and *G*. *duodenalis* commonly in a broad range of mammalian hosts [5]. Within *G*. *duodenalis*, eight species assemblages, or genotypes, are currently recognized, named from A to G. The hosts of assemblages A and B of *G. duodenalis* are the humans and other primates, livestock, domestic carnivores and wild mammals; C and D infect canids, E is common in hoofed livestock, F is typical for cats, G infects rodents and H was isolated from marine mammals [6]. The most important are zoonotic assemblages A and B, within each of them being isolated by protein polymorphisms or allozyme electrophoresis four sub-assemblages (AI, AII, AIII, AIV and BI, BII, BIII, BIV, respectively) [7, 8].

In Romania, limited data exist regarding the prevalence of *Giardia* infection in animals. Recently, the presence of *G. duodenalis* was reported in domestic carnivores; the overall prevalence was 8.5% in dogs and 27.9% in cats [9, 10]. Furthermore, assemblages A (AII), B, C (10/60; 16.7%), D (42/60; 70.0%), and E (7/60; 11.7%) have been identified in domestic and wild animals (dogs, cats, foxes, deer, wolves, raccoon dogs and muskrats) [11–13]. Consequently, the study of *Giardia* spp. infection in Romania is a field of high importance.

The long-tailed chinchillas (*C. lanigera*) are mountainous and crepuscular animals native to South America. Extensively hunted for their fur during the 19th century the species is now almost extinct in the wild, several colonies being identified only in Chile [14]. Due to their complex social behavior and attractive aspect, chinchillas became increasingly popular as pets across the world. At the same time, because of the softest, longest and finest furs among wild animals, the species became of interest for animal breeders. Farming of chinchilla dates back to 1923, when M. F. Chapman began to raise chinchillas in captivity, being the inception of what has become an industry [15]. Intensive farming exposed chinchillas to different pathogens, which are probably less common in the wild animals. Of these, water-borne parasitic diseases, particularly giardiasis, may cause clinical and sanitary problems and lead to production and economic losses [16]. Currently, there are about 75 chinchilla farms in Romania, with a production of 12,500 animals exported per year. It manifests also an increasing trend of chinchillas’ farming, whose debut in Romania dates back about 10 years ago (http://agfcicr.ro/). Due to the increasing number of farmed chinchillas in Romania, and the lack of information on the occurrence and zoonotic potential of *G*. *duodenalis* in these animals, the present study aimed to investigate the prevalence of the infection and preliminary genotyping of the isolates in Romanian chinchilla farms.

## Methods

### Animals and collection sites

Five farms with an overall stock of 5500 animals were involved in the study. Of these 2200 were breeding animals and the rest were kits and young of different ages. The following abbreviations were used for the farms studied: BM, RG, SB, SM and LU. All farms use the intensive growth closed system, but farms BM and RG also buy animals from small farmers who grow chinchillas in polyspecific farms exposed to contact with other species. A total of 341 fecal samples were collected, representing 6.2% of the stock. Of these, 171 samples were from chinchilla mothers and 170 from young animals (Table 1).

**Table 1:** Prevalence of *G*. *duodenalis* in fecal samples collected from long-tailed chinchillas in farms in Romania

Farm code	Total		Chinchilla mothers		Kits/young		Comparison	
	F	Prevalence (%) (95% CI)	F	Prevalence (%) (95% CI)	F	Prevalence (%) (95% CI)	Chi-square	*P-*value
BM	49/80	61.3 (49.7–71.9)	29/52	55.8 (41.3–69.5)	20/28	71.4 (51.3–86.8)	1.880	0.170
RG	56/80	70.0 (58.7–79.7)	24/28	85.7 (67.3–96.0)	32/52	61.5 (47.0–74.7)	5.065	0.024
SB	40/60	66.7 (53.3–78.3)	18/30	60.0 (40.7–77.3)	22/30	73.3 (54.1–87.7)	1.200	0.273
SM	19/60	31.7 (20.3–45.0)	9/30	30.0 (14.7–49.4)	10/30	33.3 (17.3–52.8)	0.077	0.781
LU	26/61	42.6 (30.0–55.9)	10/31	32.3 (16.7–51.4)	16/30	53.3 (34.3–71.7)	2.769	0.096
Total	190/341	55.7 (50.3–61.1)	90/171	52.6 (44.9–60.3)	100/170	58.8 (51.0–66.3)	1.325	0.2497

*Abbreviations*: *F* Frequency, *CI* confidence interval

### Sample processing

Each fecal sample was individually examined by flotation technique using saturated sodium chloride solution (specific gravity 1.28) [17], followed by microscopic examination (light microscopy, magnification: 10×, 20×, 40×) for the identification of *Giardia* cysts. Briefly, 0.5 g of feces/sample was homogenized with 10 ml of distilled water, filtered and centrifuged at 3000× *g* for 10 min. The supernatant was discarded, and the sediment containing *Giardia* cysts was transferred to an Eppendorf tube and used for DNA extraction.

### DNA extraction and PCR analysis

DNA extraction was performed from *Giardia*-positive samples, confirmed by microscopic examination, using Isolate Fecal DNA kit (Bioline, London, UK). To increase the specificity of DNA amplification, a semi-nested PCR reaction was performed targeting the glutamate dehydrogenase (*gdh*) gene in a T100 Thermal Cycler (Bio-Rad, Hercules, USA) [18, 19]. The PCR reaction mix contained 2× Red PCR Master mix (Rovalab, Teltow, Germany), 12 pmol of primers, 1 μl of genomic DNA; the reaction profile consisted of 1 cycle of initial denaturation at 95 °C for 5 min, followed by 40 cycles of 30 s each at 94 °C, annealing at 50 °C for 30 s for the primary reaction and 60 °C for secondary reaction, extension at 72 °C for 1 min and final extension at 72 °C for 5 min. Agarose gel (1.5%) electrophoresis stained with SYBR Safe DNA gel stain (Invitrogen, Carlsbad, USA) was performed for the visualization of PCR products.

### RFLP

For discrimination of all assemblages of *G. duodenalis*, RFLP analysis was performed using *Rsa* I and *Nla*IV (Biolabs, New England, US) restriction enzymes [18]. The amplified fragments were digested in a total volume of 50 μl, as recommended by the manufacturer’s instructions, 5 min at 37 °C for *Rsa*I, 1 h for *Nla*IV, and the reactions were stopped by 20 min of incubation at 60 °C. The digested products were visualized by electrophoresis on 3% agarose gel.

### DNA sequencing

The PCR products were purified by using QIAquick PCR purification kit (Qiagen, Hilden, Germany) and sequenced at Macrogen Europe (Amsterdam). Nucleotide sequence data from this study were submitted to the GenBank database under the accession numbers MG432793–MG432795.

### Statistical analysis

The frequency of *Giardia*-positive samples, their prevalence and 95% confidence interval were calculated. The difference in prevalence between age groups and among farms was statistically analyzed by a Chi-square test. Statistical significance was set at a *P*-value of ≤ 0.05. All statistical analyses were performed using EpiInfo software version 3.5.1. (Centers for Disease Control and Prevention: http://wwwn.cdc.gov/epiinfo/).

## Results

### The occurrence of *Giardia* spp.

*Giardia* cysts were identified in 190 of 341 (55.7%, 95% CI: 50.3–61.0) fecal samples by microscopic examination. All 190 microscopically identified *Giardia*-positive samples were positive by PCR. General prevalence recorded the highest value in farm RG (56/80, 70%, 95% CI: 58.7–79.7%) and the lowest in farm SM (19/60, 31.6, 95% CI: 20.3–45.0%) (*χ*^2^ = 28.83, *df* = 4, *P* < 0.001). The infection was somewhat more frequent in young animals (100/170, 58.8%, 95% CI: 51.0–66.3%) compared to mother chinchillas (90/171, 52.6%, 95% CI: 44.9–60.3%) but the difference was not statistically significant (*χ*^2^ = 1.09, *df* = 1, *P* = 0.25) (Table 1).

### *Giardia* spp. assemblages

In total, three *Giardia* assemblages (B, D and E) were found in the chinchilla farms studied. Assemblage B was the most prevalent (151/190, 79.5%), followed by D (33/190, 17.4%) and E (6/190, 3.1%). The identified sub-assemblages were BIV (145/190, 76.3%) and BIII (6/190; 3.1%) (Table 2).

**Table 2:** Assemblages of *G*. *duodenalis* identified by PCR-RFLP and sequencing targeting the *gdh* gene in fecal samples of long-tailed chinchillas from farms in Romania

Farm	Age	Assemblage (RFLP)		Assemblage (sequencing) (no. of samples)			
		*Nla*IV	*Rsa*I	BIII	BIV	D	E
BM	CM	BIII/BIV/E/D	BIII; BIV	3	20	4	2
	Y	BIII/BIV/D	BIV		16	4	
RG	CM	BIII/BIV/D/E	BIV		19	3	2
	Y	BIII/BIV/D/E	BIV	1	22	7	2
SB	CM	BIII/BIV/D	BIV		15	3	
	Y	BIII/BIV/D	BIII; BIV	2	17	3	
SM	CM	BIII/BIV/D	BIV		4	5	
	Y	BIII/BIV	BIV		10		
LU	CM	BIII/BIV/D	BIV		8	2	
	Y	BIII/BIV/D	BIV		14	2	
Total				6	145	33	6

*Abbreviations*: *CM* chinchilla mothers, *Y* young

Sequence analysis of fecal samples confirmed the infection with *G. duodenalis* sub-assemblages BIII, BIV, D and E (Table 2). Assemblages B (MG432795) and D (MG432793) were common in all farms in the study, in both age categories, and Assemblage E (MG432794) was identified only in farms BM and RG. No mixed assemblage infections were detected in animals in this study.

## Discussion

The study of intestinal parasites in the long-tailed chinchilla is an important field of interest due to a permanent contact of this pet or farmed animal with humans. Among parasitic diseases identified in this species, giardiasis seems to be the most significant, due to the zoonotic character and increased values of prevalence reported worldwide (Table 3). The prevalence revealed in the present study (55.7%) is slightly increased compared to that reported in farmed chinchilla from other regions (8.0–38.0% in Brazil, 34.4% in Argentina and 39.4% in Italy) but is comparable to those reported in pet animals (10.0–92.3%).

**Table 3:** Reported prevalence of *Giardia* spp. infection in the long-tailed chinchilla

Country	Husbandry system (pet/farmed/wild)	Prevalence (%)	Frequency	Detection method	Reference
Argentina	Farmed	34.42	84/244	Wet mounts, IFA	[46]
Belgium	Pet	66.3	53/80	SCF	[24]
Brazil	Farmed	8.0	20/250	ZCF	[47]
Brazil	Farmed	38.0	38/100	ZCF	[48]
Brazil	Pet	10.0	6/60	ZCF	[49]
Brazil	Farmed	31.37	80/255	ZCF	[28]
Chile	Wild	Negative	na		–
China	Pet	37.5	36/96	SF	[50]
China	Pet	27.1	38/140	PCR	[51]
Europe	Pet	61.4	326/531	ELISA	[31]
Italy	Farmed	39.4	41/104	DFA	[31]
Portugal	Pet	35.2–92.3	na	ZCF, SF	[52]
Russia	Pet	50.0	25/50	CFM	[53]
Russia	Pet	Positive	na	ANF	[29]
Peru	Wild	Negative	na		–
Romania	Farmed	55.7	190/341	NaClF	Present study

*Abbreviations*: *ANF* ammonium nitrate flotation, *CFM* combined flotation method, *DFA* direct fluorescent assay, *ELISA* enzyme-linked immunosorbent assay, *IFA* immunofluorescence assay, *na* not applicable, *NaClF* sodium chloride flotation, *SCF* sucrose gradient centrifugation-flotation technique, *SF* sugar flotation (Sheather’s sugar solution), *ZCF* zinc-sulfate centrifugation-flotation

The discrepancies of prevalence recorded in existing studies can be explained by the different diagnostic value of copromicroscopic methods used, determined by the technique, less than the density of supersaturated solutions [20]. Moreover, coproscopic techniques have a lower diagnostic value, the prevalence determined by other modern serological or molecular methods (ELISA, IFA, PCR) being 2.6-fold higher in dogs and 3.7-fold higher in cats [21]. As such, we consider that the prevalence of infection revealed in this study, although high, can be appreciated as undervalued.

Prevalence of *Giardia* infection is generally influenced by many factors, such as the sensitivity of the diagnostic method used, the peculiarities of the biological cycle of the parasite (the discontinuities of cysts removal), the host, the age of host, the growth system, and the hygiene conditions (water, food, bedding) [22]. A variety of factors favor the emergence and transmission of infection in chinchilla populations. These risk factors may differ among pet and farmed chinchilla. Regarding pet chinchilla, participation in shows and contact with other pet animals, such as dogs, cats or other rodents, are significant [23, 24]. In farmed chinchillas, the age of animals, stress, poor husbandry system associated with low quality of water source, overcrowding and close contact with feces seems to act as predisposing factors. Juvenile chinchillas are more sensitive to acquire the infection [25]. Intensive rearing in plastic or metal cages, with fecal accumulation underneath and vulnerability of the drinking-water-processing system, favor the contact between animals and cysts of *Giardia* spp. [26, 27]. Captivity associated with specific stress emphasizes the sensitivity of chinchilla to *G. duodenalis* infection, an aspect demonstrated by the absence of *Giardia* spp. infection in wild animals [28, 29].

Chinchillas harbor various assemblages (A, B, C, D and E) of *G. duodenalis*, representing a potential zoonotic risk (Table 4). Assemblage B is the most common, being identified in almost all reported studies, except for an axenic isolate of *G*. *duodenalis* from Germany [30], in which assemblage A was identified. In our research, RFLP analysis of *G. duodenalis*-positive samples revealed a high occurrence of assemblage B isolates grouped into sub-assemblages BIII and BIV, representing the main assemblages involved in chinchilla’s infection. In the present study, no mixed assemblage infections were detected, similar to previous studies [16, 31]. However, our data do differ from those reported in Belgium and Germany, which showed the presence of mixed assemblage A, B, C and E infections in chinchillas [24, 32].

**Table 4:** Assemblages of *G. duodenalis* identified in the long-tailed chinchillas

Country	Type of animal (pet/farmed)/assemblage		Reference
	Pet	Farmed	
Austria	B	–	[32]
Belgium	A, AI, AII, B, BIV, C, E	–	[24]
Brazil	B	–	[16]
Brazil	BIV	–	[54]
China	AI, AII, BIV, BIV-1, BIV-2	–	[51]
Croatia	B	–	[55]
Czech Republic	B	–	[56]
Germany	A	–	[30]
Germany	A, B, D	–	–
Italy	–	B, C	[31]
Romania	–	BIII, BIV, D, E	Present study

The presence of C and D assemblages typical for canids, and E from hoofed livestock, in chinchillas is quite interesting. In this work, the existence of assemblages E in farms BG and RG can be explained by acquiring animals from farms in which ruminants were also kept, the direct or indirect contact between the two species being possible.

Generally, multiple factors can explain the diversity of assemblages identified across the world. Interspecies transmission is of particular importance for the zoonotic risk of infection, domestic animals being the source of human infection. Reverse or cross-species transmission of different assemblages (BIV, E) has also been demonstrated in areas where humans, primates and livestock overlap in their use of habitat [33]. Interspecific transmission is possible between species belonging to different taxa, from rodents to carnivores and from ruminants to humans [34]. It is also proven that *G*. *duodenalis* from the North American beaver (*Castor canadensis*) may infect Mongolian gerbils (*Meriones unguiculatus*); in this case, the transmission was carried out between two rodent species [35]. Transmission of *Giardia* spp. between different species of rodents is also confirmed in other older studies [36]. Nevertheless, Goltz [37] demonstrated that *G. chinchillae* from *C. lanigera* were not infective to laboratory mice, rats and guinea pigs. However, the interspecies transmission may explain the presence of assemblages D and E in our study, sustained by the existence of guard dogs and small ruminants in the examined farms.

Transport vectors can also play a significant role in the transmission of giardiasis [38]. It is confirmed that assemblage E of *G*. *duodenalis* is carried by flies, increasing the possibility of repeated infection or cross-transmission between sensitive species, by mechanical transmission [39]. As a result, despite of the strong host specificity and narrow host range of assemblage E, which is mostly identified in cloven-hoofed mammals, the involvement of the transport hosts can ensure the transmission of this assemblage to captive chinchillas [8].

Water source is also important in the circulation of *G*. *duodenalis* cysts, giardiasis being recognized as one of the major waterborne diseases [40]. Although the long-tailed chinchilla is a species adapted to aridity, with low water needs, it prefers the open dish drinker [41]. The best water supply in chinchilla farming is represented by bottled water, free of pathogens and chlorine [42]. Tap and well water are also accepted sources, but they present the risk of contamination with *Giardia* cysts. Surprisingly, in Romania, bottled water seems to have an increased risk of infection compared with wells or tap water [43]. Feces of different animal species can pollute water sources, shedding cysts into the water supply [44]. These cysts can pass through water treatment, even for pristine or filtered drinking water. Furthermore, *Giardia* spp. cysts have a demonstrated effective resistance to chlorination [45]. Tap water was the source in studied farms, without an additional water filtration; chlorination and filtration performed by water plant suppliers being the unique treatments. Combining predisposing factors as interspecific transmission, the possible involvement of vectors and deficiencies in water supply, the increased prevalence of *G*. *duodenalis* infection in farmed chinchilla from Romania may be explained.

## Conclusions

This study revealed the increased prevalence of infection with *G*. *duodenalis* in farmed chinchilla from Romania and the presence of BIII, BIV, D and E assemblages. Further studies are needed to clarify the zoonotic risk for the owners and workers in chinchilla husbandry.

## Abbreviations

BM: Baia Mare
LU: Luncani
RG: Reghin
SB: Suceava-Bejenaru
SM: Suceava-Morozan
